# Enhanced cleaning strategies for UF/DF membranes in biopharmaceutical downstream processing

**DOI:** 10.1186/s40643-026-01045-0

**Published:** 2026-04-02

**Authors:** Xinye Han, Zhenhao Bian, Xuyue Han, Huaguang Wang, Han Liu, Yajuan Zhou, Yiqing Cui, Xumei Liu

**Affiliations:** Downstream Process Development (DSPD), WuXi Biologics, No.200 Meiliang Road, Binhu District, Wuxi, 214092 Jiangsu China

**Keywords:** Ultrafiltration/diafiltration (UF/DF), Membrane fouling, Cleaning strategies, Permeate-closed cleaning, Backwashing, Biologics downstream processing

## Abstract

Membrane fouling during ultrafiltration/diafiltration (UF/DF) remains a major challenge for extending membrane lifetime in biologics manufacturing. Developing cleaning strategies that combine high efficiency with scalability is essential for robust downstream processing. In this study, different UF/DF membrane cleaning methods were assessed under bench-scale conditions with consideration for large-scale implementation. Permeate-closed cleaning (PCC), which temporarily closes the permeate port to enhance shear, effectively mitigated normalized water permeability (NWP) decline. Furthermore, forward and reverse PCC at elevated feed flux achieved comparable fouling control with fewer steps by leveraging hydrodynamic shear and localized backwashing. To simplify large-scale adoption, high-flux PCC was developed and demonstrated the ability to restore NWP and maintain stability over extended cycles. Quantitatively, high‑flux PCC reduced early NWP decay by ~ 18% over the first three cycles and recovered > 15% NWP in previously fouled membranes, stabilizing permeability over extended reuse. Backwashing under controlled negative transmembrane pressure (TMP) further improved pore-level cleaning and extended membrane usability. Additionally, combining sodium hypochlorite (NaClO) with sodium hydroxide (NaOH) provided strong oxidative and alkaline action; within the evaluated range (25–150 ppm NaClO), 50 ppm was identified as a practical lower limit for effective recovery, while 150 ppm restored NWP from ~ 70 to ~ 100% with residual NaClO < 0.02 ppm after final rinsing. Collectively, these findings present a portfolio of cleaning strategies—hydrodynamic, chemical, and combined approaches—that enhance cleaning efficiency, reduce chemical exposure, and support sustainable UF/DF operations in biologics manufacturing.

**Graphical abstract:**

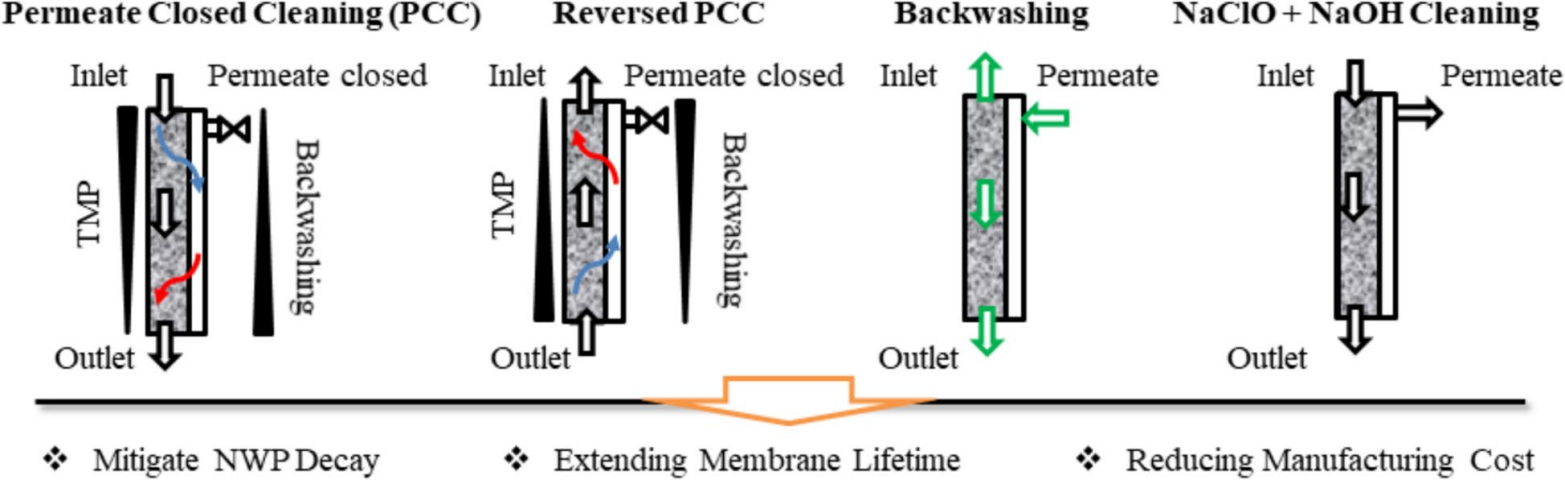

**Supplementary Information:**

The online version contains supplementary material available at 10.1186/s40643-026-01045-0.

## Introduction

Monoclonal antibodies (mAbs) represent the largest and fastest-growing class of biotherapeutics, widely used for the treatment of cancer, autoimmune disorders, and inflammatory diseases (Carter et al. 2022; Buss et al. 2012; Shepard et al. 2017; O’Mahony et al. 2006). Their clinical success and market dominance have driven the development of high-concentration formulations to enable subcutaneous administration, which introduces significant challenges in downstream processing (Desai et al. 2023; Li et al. 2014). Ultrafiltration/diafiltration (UF/DF) is a critical tangential-flow filtration (TFF) unit operation in the final formulation, enabling buffer exchange and volume reduction while maintaining product quality (Whitaker et al. 2022; Baek et al. 2017). As mAb titers and formulation concentrations increase, robust UF/DF performance and effective membrane cleaning become essential for process consistency and cost control. Moreover, upstream media components can directly modulate critical quality attributes such as mAb charge heterogeneity, further linking formulation targets to downstream robustness requirements (e.g., UF/DF cleaning and reuse), as evidenced by uridine‑driven shifts in acidic/basic variants in CHO fed‑batch cultures (Niu et al. 2018).

Separation unit operations such as ultrafiltration, nanofiltration, and diafiltration (UF/NF/DF) play essential roles in fractionating and conditioning complex biological feedstocks due to their scalable hydrodynamics, tunable selectivity, and low thermal stress. Integrated approaches—including UF/DF sequences, UF/NF cascades, and hybrid membrane–chromatography arrangements—are increasingly used to decouple size‑ and interaction‑based separations (Chen et al. 2023; Rajendran et al. 2021). Reviews in natural‑product systems (e.g., mangiferin and carminic acid) further highlight extraction and purification challenges, solvent/condition trade‑offs, and the need for robust and cleanable unit operations (Castro‑Muñoz et al. 2024; Ferreyra‑Suarez et al. 2024). These insights underscore the importance of reliable membrane steps within broader process‑intensification strategies. Against this background, our study examines enhanced cleaning strategies for PES UF/DF to support reusability and process consistency under industry‑relevant soils. Beyond biotherapeutic feeds, bioresource‑derived adsorbents exemplify how interface engineering can improve aqueous‑phase separations and mitigate organic loading/fouling risks; for instance, tailored oil‑palm‑trunk biochar achieved efficient dye adsorption via controlled carbonization that tunes surface chemistry and pore accessibility (Hakimi et al. 2026).

Membrane fouling in UF/DF arises from adsorption, deposition, gel layer formation, and concentration polarization at the membrane interface, leading to reduced permeability and selectivity (D’Souza et al. 2005; Shi et al. 2014). Cleaning effectiveness is commonly assessed by normalized water permeability (NWP) measured under standard conditions, which serves as a reproducible benchmark for cassette cleanliness across cycles (Cheryan 1998; Samavedam et al. 2007). In addition, because our study includes both parallel control comparisons and before‑and‑after cleaning evaluations, normalized water permeability (NWP) was used as the unified quantitative indicator of membrane fouling and permeability recovery.

Beyond process‑side controls, PES/PSF‑based UF materials now leverage zwitterionic designs and PDA‑enabled interfaces to intrinsically curb protein fouling and stabilize flux, while recent advances with ZIF‑8–modified CA and PA–CA composite layers further strengthen surface properties and fouling resistance (Li et al. 2021; Huang et al. 2022; Mulyati et al. 2020; Zarghami et al. 2019). Recent studies indicate that incorporating ZIF‑8–modified cellulose acetate architectures and designing polyamide–cellulose acetate thin‑film composite layers can markedly improve surface attributes and enhance fouling resistance in membrane systems (Vatanpour et al. 2023; Ounifi et al. 2022).

Several cleaning strategies for UF/DF membranes have been reported in biologics manufacturing because high protein concentrations and repeated cassette reuse increase fouling risk and impact process economics (D’Souza et al. 2005; Shi et al. 2014). Reported approaches fall into two categories: physical and chemical. Physical methods, such as backwashing and flow reversal, aim to dislodge loosely attached foulants and alleviate reversible fouling, often serving as intermediate steps between chemical cycles (Cui et al. 2018; Yang et al. 2023a, b, c). Chemical cleaning relies on agents that disrupt or solubilize foulants; NaOH is widely used in bioprocessing for its strong alkalinity, which promotes protein hydrolysis and saponification of lipids, while NaClO combined with NaOH can enhance removal of organic and biofouling contaminants through oxidative degradation (Krack. 1995; Wu et al. 2006). Beyond alkaline/oxidative routes, solvent use is also documented in adjacent workflows: for instance, ethanol may be applied as a rinsing medium under strict handling and pre‑filtration steps when processing microplastic‑containing water samples (Yang et al. 2023a, b, c). More broadly, material composition and processing can modulate particulate release and fouling propensity—as observed for biodegradable versus conventional polymers and for additively manufactured membranes—reinforcing our strategy of linking cleaning choices to foulant/material characteristics in industrial UF/DF (Yang et al. 2023a, b, c; Pini Pereira et al. 2025). In addition, enzymatic cleaning agents (e.g., proteases) are also employed for proteinaceous soils and can reduce harsh chemical exposure when properly matched to the foulant spectrum. However, enzymatic cleaning is not used as the primary route for antibody industry due to (i) the need for broad foulant coverage and straightforward scale‑up/verification in UF/DF operations, (ii) PES–NaOH compatibility within bounded exposure windows, and (iii) GMP implement ability considerations for enzyme residual clearance/validation (Gitis 2014; Merck 2026).

Polyethersulfone (PES) membranes, the focus of this study, are compatible with these strategies due to their broad chemical tolerance and stability under alkaline and oxidizing conditions (Marino et al. 2018; Arkhangelsky et al. 2007). Previous work by Baek et al. demonstrated the effectiveness of permeate-closed cleaning with reverse flow using deionized water for regenerated cellulose membranes, highlighting the potential of hydrodynamic cleaning cycles as an alternative to aggressive chemicals (Baek et al. 2018). Permeate‑closed cleaning (PPC) operates by closing the permeate outlet to force all feed flow along the membrane surface, thereby increasing shear stress, reversing local transmembrane pressure profiles, and promoting foulant detachment through controlled surging or backflow events. This approach has been applied in TFF, microfiltration, and wastewater membrane systems as a low‑chemical hydrodynamic cleaning mode capable of mitigating reversible fouling and restoring flux without structural damage to the membrane (Baek et al. 2018; Kim et al. 2020; Shi et al. 2014). Such PPC‑based strategies offer a promising complement to chemical cleaning, particularly for systems requiring reduced chemical consumption or extended membrane lifetime.

Building on this concept, our study focuses on PES membranes, which are widely used in biologics UF/DF processes, and introduces additional strategies developed with scalability considerations. Specifically, we integrate high-flux permeate-closed operation, forward/reverse surging, backwashing under negative Transmembrane Pressure (TMP), and low-dose NaClO–NaOH combinations. This work aims to develop and evaluate UF/DF membrane cleaning strategies that not only improve cleaning efficiency but also enhance process sustainability by reducing membrane waste and chemical consumption. By linking hydrodynamic and chemical approaches to foulant-specific mechanisms, we provide parameterized guidance for scalable and resource-efficient bioprocessing.

## Materials and methods

### Materials

Three highly purified monoclonal antibodies (mAb A, mAb B, and mAb C), all derived from CHO-K1 cells and were produced at WuXi Biologics as small‑scale development batches, were used in UF/DF experiments. Pellicon® XL cassettes (Millipore, cat. PXB030A50) with an internal A-screen and 30 kDa PES membranes were employed in all trials. These cassettes use a flat-sheet configuration and provide an effective membrane area of 0.005 m^2^.

### Ultrafiltration/diafiltration procedure

**Fig. 1:**
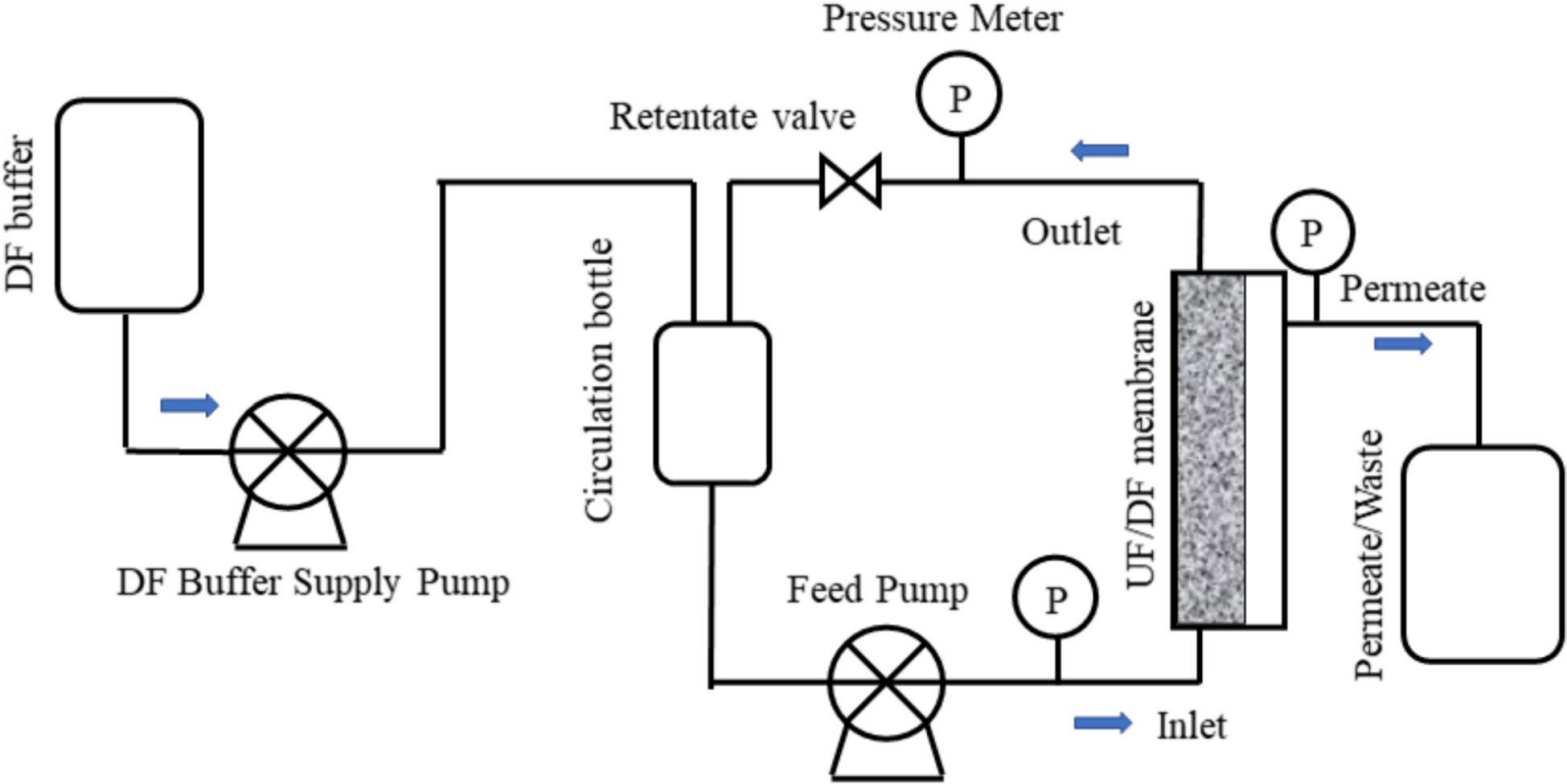
Apparatus configuration for ultrafiltration and diafiltration experiments

The UF/DF setup is shown in Fig. 1. Flow was driven by a peristaltic pump, and silicone tubing connected system components. Inlet, outlet, and permeate pressures were monitored using pressure gauges, and TMP was controlled via the retentate valve. Except for backwashing studies, flow directions are indicated by blue arrows in Fig. 1. Typical UF/DF steps are illustrated in Table 1.

**Table 1:** Process flow for four experimental groups

UF/DF steps	Control	PCC	0.1 M NaOH soaking	Combination
PCC (HPW)		x		x
Rinse1(HPW)	x	x	x	x
PCC (NaOH)		x		x
Sani (NaOH)	x	x	x	x
PCC (HPW)		x		x
Rinse2(HPW)	x	x	x	x
Pre-use NWP testing		x		x
EQ/UF/DF/OC/recirc	x	x	x	x
PCC (EQ&HPW)		x		x
Rinse3 (HPW)	x	x	x	x
PCC (NaOH)		x		x
0.1 M NaOH soaking				x
Sani. (NaOH)	x	x	x	x
PCC (HPW)		x		x
Rinse4 (HPW)	x	x	x	x
Post-use NWP testing		x		x
Storage	x	x	x	x

Process durations were recorded per run and are provided in the Supplementary Information (Figs. S1–S5), as UF/DF was controlled by target concentration, diavolumes, and over‑concentration endpoints rather than fixed time. Unless otherwise noted, solution volumes are reported normalized to membrane area (L/m^2^): routine rinse/sanitization/equilibration/storage steps used 20 L/m^2^ per step; diafiltration used 5 DV for mAb A and mAb C, and 6 DV for mAb B.

Protein concentration in the permeate was measured for all evaluated cycles and remained below detection limit, indicating no loss of retention or membrane leakage across cleaning conditions.

Pressure at the inlet, outlet, and permeate was monitored using pressure gauges, and the TMP was controlled via a retentate valve. Blue arrows indicate flow directions during UF/DF experiments, except in the backwashing study.

Monoclonal antibody A was used in all studies, except those involving NaClO cleaning. The control group operated under the following parameters: feed flux of 200 Liter/Square Meter/Hour (LMH); 20 L/m^2^ flush for each step (rinse, sanitization, equilibration, storage); 1 M NaOH recirculated for 60 min during sanitization; highly purified water (HPW) for rinses; and 0.1 M NaOH as storage buffer. Loading was 300 g/m^2^, TMP was maintained at 1.2 bar during sample processing, UF target concentration was 50 g/L, diafiltration used 20 mM histidine buffer (pH 6.0) for 5 Diavolumes (DV), and the Over Concentration (OC) step targeted 75 g/L.

For studies involving 150 ppm NaClO cleaning, mAb B was used with feed flux of 300 LMH, loading of 400 g/m^2^, TMP at 0.7 bar, UF target concentration of 50 g/L, diafiltration buffer of 20 mM histidine with 7.5% sucrose (pH 6.0) for 6 DV, and OC target of 60 g/L. For studies with 25 and 50 ppm NaClO cleaning, mAb C was used following the same parameters as mAb A.

### Permeate-closed cleaning (PCC) and 0.1 M NaOH soaking

Four experimental groups were evaluated: (i) control, (ii) PCC, (iii) 0.1 M NaOH soaking, and (iv) a combination of both (PCC + 0.1 M NaOH soaking). In PCC, the permeate port was closed and the retentate outlet fully opened, with feed flux at 200 LMH. This step, using the corresponding buffer, was applied before all rinse and sanitization steps (Table 1). In NaOH soaking, permeate-closed cleaning was performed with 0.1 M NaOH, followed by pump shutdown to allow soaking for 3 h prior to post-sanitization. The combination group integrated both approaches.

### Forward and reverse PCC at higher feed flux

Two experimental cleaning modes were evaluated (Table 2). Forward cleaning directed flow from inlet to outlet, while reverse cleaning directed flow from outlet to inlet. In both cases, the permeate port was closed. Each step consisted of flushing 20 L/m^2^ at twice the standard feed flux (400 LMH), followed by 30 min circulation. These steps were performed prior to Rinse 1 and during pre-use and post-use sanitization.

**Table 2:** Process flow comparison between control and F&RPCC groups

UF/DF steps	Control	F&RPCC
F&RPCC (HPW)		x
Rinse1(HPW)	x	x
F&RPCC (NaOH)		x
Sani. (NaOH)	x	x
Rinse2(HPW)	x	x
Pre-use NWP testing	x	x
EQ/UF/DF/OC/recirc	x	x
Rinse3 (HPW)	x	x
F&RPCC (NaOH)		x
Sani. (NaOH)	x	x
Rinse4 (HPW)	x	x
Post-use NWP testing	x	x
Storage	x	x

### PCC at higher feed flux

Two groups were evaluated (Table 3). To generate fouled membranes, loading was 500 g/m^2^ for the first two cycles, then 300 g/m^2^ for subsequent cycles. In the high-flux cleaning group, HPW permeate-closed cleaning was performed at 400 LMH for 20 L/m^2^ before Rinse 2, Rinse 3, and Rinse 4. Sanitization was modified: permeate-closed cleaning with 1 M NaOH (20 L/m^2^ at 400 LMH) followed by 30 min retentate circulation, then the conventional 1 M NaOH flush (20 L/m^2^) with 30 min full circulation.

**Table 3:** Process flow comparison between control and PCC groups

UF/DF steps	Control	PCC
Rinse1(HPW)	x	x
PCC (NaOH)		x
Sani. (NaOH)	x	x
PCC (HPW)		x
Rinse2(HPW)	x	x
Pre-use NWP testing	x	x
EQ/UF/DF/OC/recirc	x	x
PCC (HPW)		x
Rinse3 (HPW)	x	x
PCC (NaOH)		x
Sani. (NaOH)	x	x
PCC (HPW)		x
Rinse4 (HPW)	x	x
Post-use NWP testing	x	x
Storage	x	x

### Backwashing

The backwashing procedure is shown in Table 4. UF/DF cycles were performed until post-use NWP decreased to 51% of its initial value. Backwashing without sample treatment was applied twice, followed by NWP testing, then four cycles with mAb treatment. Modifications included:

**Table 4:** Process flow for the backwashing group compared with the control

UF/DF steps	Control	Backwashing
Rinse1(HPW)	x	x (Backwashing integrated)
Sani. (NaOH)	x	x (Backwashing integrated)
Rinse2 (HPW)	x	x
Pre-use NWP testing	x	x
EQ/UF/DF/OC/recirc	x	x
Rinse3 (HPW)	x	x (Backwashing integrated)
Sani. (NaOH)	x	x (Backwashing integrated)
Rinse4 (HPW)	x	x
Post-use NWP testing	x	x
Storage	x	x

Rinse: HPW flushed at 200 LMH from inlet with outlet and permeate open for 20 L/m^2^, then from permeate with inlet and outlet open for 20 L/m^2^ while maintaining 0.7 bar permeate pressure.

Sanitization: 1 M NaOH flushed at 200 LMH from inlet with outlet and permeate open for 20 L/m^2^, followed by 30 min recirculation; then flushed from permeate under the same conditions, followed by another 30 min recirculation.

### Combination of NaClO and NaOH cleaning

Groups using 150 ppm NaClO and 0.5 M NaOH were processed as described in Ultrafiltration/Diafiltration Procedure. Two UF/DF cycles were first performed to generate fouled membranes, which were then cleaned using 150 ppm NaClO followed by 0.5 M NaOH. Similar experiments were conducted for 50 ppm and 25 ppm NaClO combined with 0.5 M NaOH. Cleaning was applied to membranes with NWP reduced to below 60% of the initial value.

### Normalized water permeability testing

NWP was measured at pre-use and post-use of each cycle with the system filled with HPW. Pump speed and retentate valve were adjusted to maintain inlet pressure at ~ 0.7 bar and outlet pressure at ~ 0.3 bar. After stabilization for 5–10 min, permeate flow rate was recorded three times along with water temperature. NWP was calculated as:

$$ {\mathrm{NWP}} = \frac{{{\mathrm{R}} \times {\mathrm{F}}}}{{{\mathrm{A}} \times \left\{ {\left( {\frac{{{\mathrm{Pin}} + {\mathrm{Pout}}}}{2}} \right) - {\mathrm{Pp}}} \right\}}} $$

where R is permeate flow rate, F is temperature correction factor, A is membrane area, Pin is inlet pressure, Pout is outlet pressure, and Pp is permeate pressure. The pre-use NWP of the first cycle was defined as the initial NWP.

## Results

### Effect of permeate-closed cleaning and NaOH soaking on NWP decay across multiple cycles

To evaluate strategies for mitigating fouling, four experimental groups were compared: control, NaOH soaking, permeate-closed cleaning (PCC), and their combination. Table 1 outlines the process modifications, where permeate-closed cleaning steps were inserted before rinse and sanitization stages, and NaOH soaking was introduced prior to post-use sanitization. Permeate-closed cleaning and NaOH soaking were intended to enhance foulant removal through two complementary mechanisms: hydrodynamic shear and flow redistribution during permeate closure, and chemical dissolution of residual deposits during extended NaOH exposure.

**Fig. 2:**
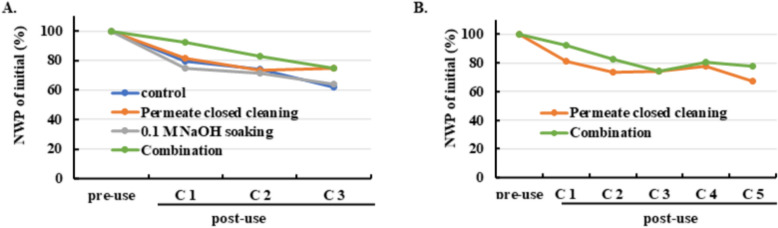
Effect of permeate-closed cleaning and NaOH soaking on NWP decay. **A** NWP decay profiles (% of initial) for the four groups over three UF/DF cycles. Initial NWP was defined as the pre-use value before cycle 1. **B** Extended NWP decay profiles for permeate-closed cleaning and combination groups over five cycles

NWP trends over three UF/DF cycles (Fig. 2A) revealed that the control and NaOH soaking groups exhibited similar declines, indicating that short-term NaOH exposure alone was insufficient to prevent fouling. In contrast, permeate-closed cleaning reduced NWP decay, and the combination of both approaches provided the greatest benefit, maintaining approximately 10% higher NWP than the control after three cycles. Extended testing for the two most effective strategies (Fig. 2B) confirmed that both continued to suppress NWP decline, with the combination outperforming permeate-closed cleaning alone. This suggests a synergistic effect: hydrodynamic shear likely removes loosely attached foulants, while NaOH soaking facilitates chemical dissolution of residual deposits.

### Effect of forward and reverse permeate-closed cleaning at high feed flux on NWP recovery

High feed flux enhances turbulence and shear forces, accelerating foulant removal from membrane surfaces and pores. It also creates a pressure gradient along the membrane length, generating localized backwashing—reverse flow from the permeate side to the retentate side—that helps dislodge embedded deposits (Arkhangelsky et al. 2007; Cui et al. 2018). Table 2 illustrates this mechanism: forward flow produces higher TMP at the inlet and localized negative TMP at the outlet, while reverse flow shifts this effect to the inlet, enabling alternating backwashing across the entire membrane.

**Fig. 3:**
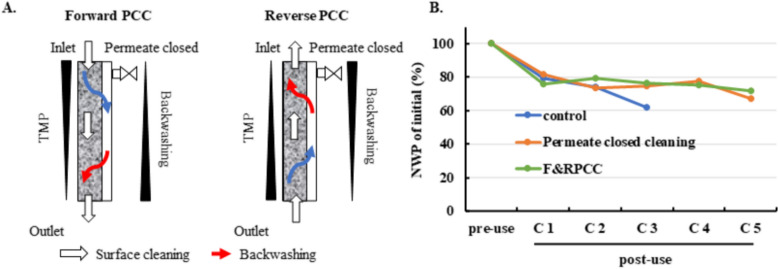
Effect of forward and reverse permeate-closed cleaning at high feed flux on NWP decay. **A** Schematic illustrating the hydrodynamic mechanism of forward and reverse permeate-closed cleaning. Forward flow (inlet to outlet) creates higher TMP at the inlet and localized negative TMP at the outlet, inducing backwashing near the outlet region. Reverse flow (outlet to inlet) shifts this effect to the inlet region. Blue arrows indicate primary flow; red arrows indicate backwashing flow. **B** NWP decay profiles (% of initial) for three groups: control, standard permeate-closed cleaning, and F&RPCC. Initial NWP was defined as the pre-use value before cycle 1

To leverage these hydrodynamic effects, forward and reverse permeate-closed cleaning (F&RPCC) was implemented at twice the standard feed flux (400 LMH) before Rinse 1 and during sanitization. Figure 3A compares the process flow of the control and F&RPCC groups, highlighting the integration of high-flux forward and reverse cleaning steps. NWP profiles (Fig. 3B) show that both permeate-closed strategies mitigated NWP decay compared with the control, but F&RPCC achieved slightly higher retention than standard permeate-closed cleaning, despite requiring fewer intermediate steps. This improvement likely reflects the combined effects of increased shear and localized backwashing under high flux.

Collectively, these findings demonstrate that F&RPCC offers a practical and scalable cleaning strategy, enhancing foulant removal through hydrodynamic mechanisms while simplifying the cleaning workflow for industrial applications.

### Effect of high-flux permeate-closed cleaning on NWP recovery and membrane longevity

Reverse permeate-closed cleaning, although effective, is operationally complex and less practical for large-scale manufacturing. Therefore, permeate-closed cleaning at higher feed flux (PCC) using the conventional flow direction was evaluated as a simplified alternative (Table 3). PCC incorporated high-flux cleaning steps before Rinse 2, Rinse 3, and Rinse 4, and modified sanitization steps combining permeate-closed cleaning at 400 LMH with traditional flow-path cleaning.

**Fig. 4:**
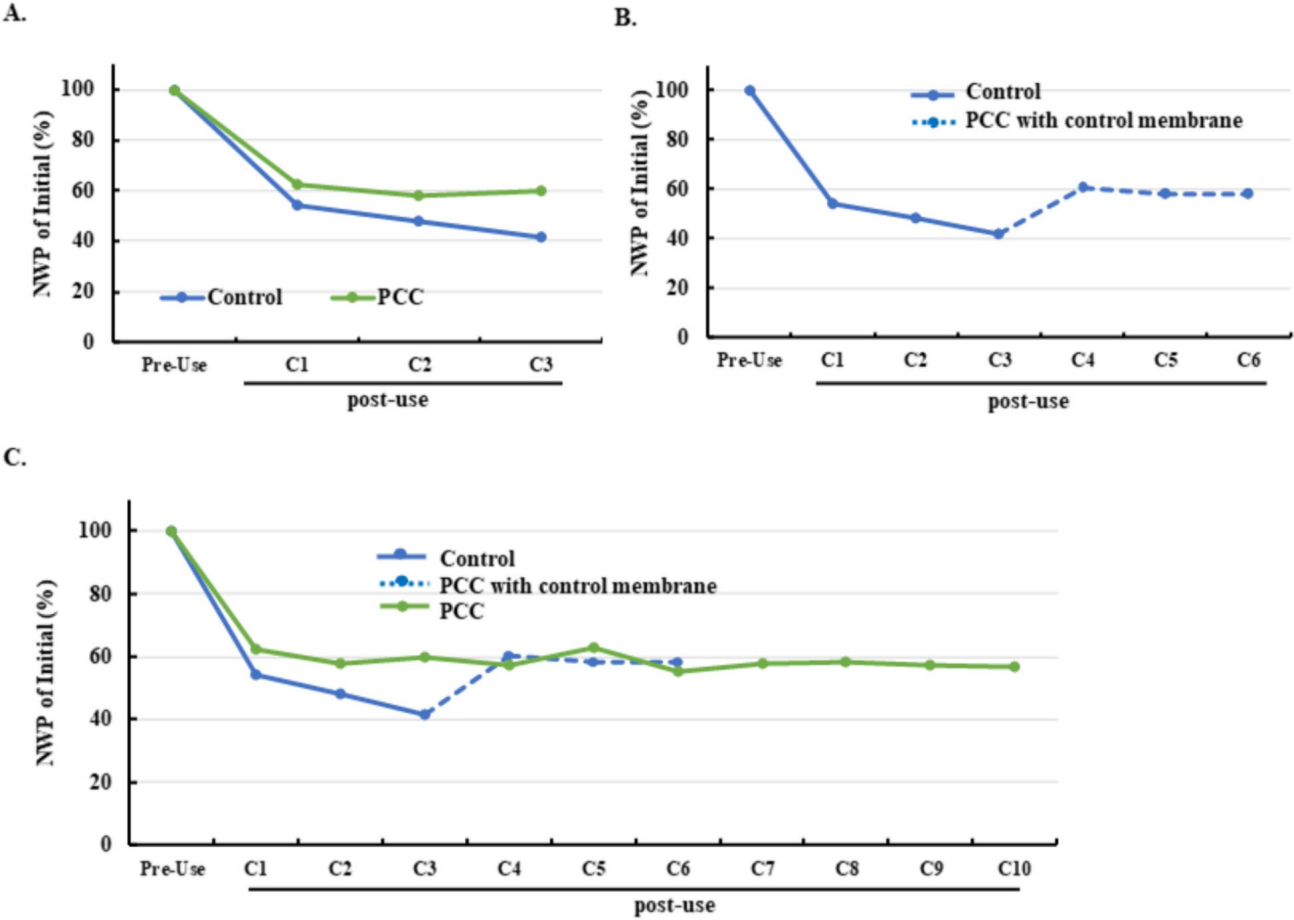
Effect of high-flux permeate-closed cleaning (PCC) on NWP decay and membrane recovery. **A** NWP decay profiles (% of initial) for control and PCC groups over three UF/DF cycles. Initial NWP was defined as the pre-use value before cycle 1. **B** Recovery of fouled membranes using PCC. Membranes cleaned by the control method for three cycles were subsequently treated with PCC for three additional cycles. **C** Extended NWP profiles for PCC and control groups over 10 cycles. Green lines represent PCC applied throughout; blue solid lines represent control; blue dashed lines represent membranes switched to PCC after initial fouling

Compared with the control, PCC reduced NWP decay by approximately 18% during the first three cycles (Fig. 4A). Unlike the control group, which exhibited progressive NWP decline, PCC maintained stable NWP after the first cycle. To assess recovery capability, membranes from the control group after three cycles were subjected to PCC and reused for three additional cycles. PCC restored more than 15% of NWP and stabilized performance through cycles 4–6 (Fig. 4B). Extended testing up to 10 cycles confirmed that PCC maintained NWP at levels comparable to early cycles, demonstrating robustness for repeated use (Fig. 4C). Furthermore, membranes rescued by PCC achieved NWP comparable to those continuously cleaned with PCC, reinforcing its effectiveness in recovering fouled membranes.

Collectively, these findings suggest that PCC offers a scalable, industry-friendly solution for mitigating NWP decay, restoring fouled membranes, and extending membrane lifetime without additional process complexity.

### Backwashing for NWP recovery and membrane longevity

Backwashing applies a negative TMP to reverse flow from the permeate side to the retentate side, dislodging foulants trapped within membrane pores (Arkhangelsky et al. 2007). Previous studies have shown that backwashing can outperform conventional forward flushing (Cui et al. 2018). To evaluate its effectiveness, a backwashing protocol was implemented in parallel with the control group (Table 4). Millipore UF/DF cassettes tolerate moderate negative TMP; preliminary data confirmed that exposure to 0.7 barwith 1 M NaOH for 72 h did not compromise membrane integrity (data not shown). Therefore, 0.7 bar was selected for this study.

**Fig. 5:**
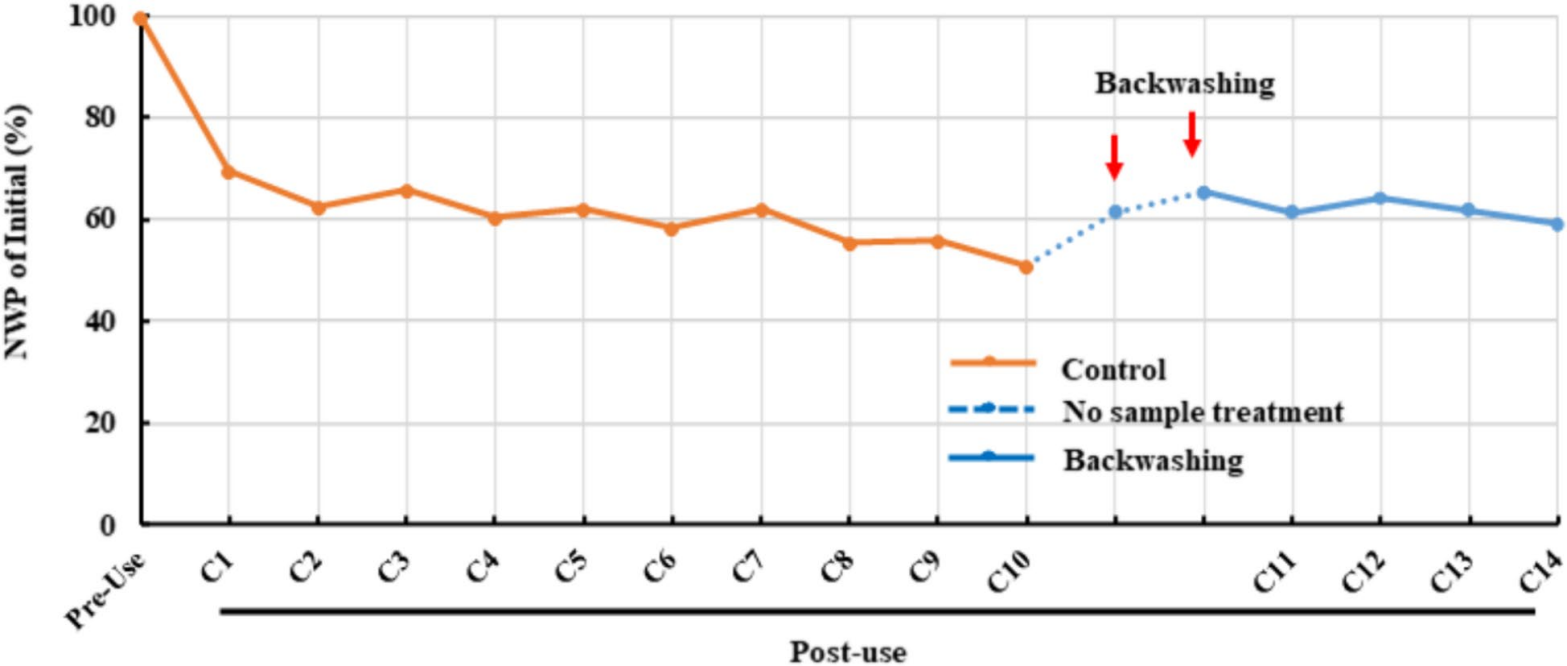
Effect of backwashing on NWP recovery and membrane lifetime. NWP profiles for membranes cleaned by the control method (orange lines) and by backwashing (blue lines) over multiple UF/DF cycles

Backwashing steps were incorporated after Rinse 1, Rinse 3, and during pre-use and post-use sanitization, preceded by conventional flushing to remove surface deposits. Multiple UF/DF cycles were performed until post-use NWP declined to 51% of its initial value, after which backwashing was applied. A single backwashing cycle restored approximately 10% of NWP, and a second cycle added an additional 4%, indicating cumulative benefits (Fig. 5). Subsequent UF/DF cycles using backwashing maintained improved NWP, with cycle 14 achieving 10% higher NWP than cycle 10. These findings highlight backwashing as an effective strategy for recovering fouled membranes and extending UF/DF membrane lifetime, offering a practical option for long-duration manufacturing campaigns.

### Efficiency of combined NaClO and NaOH cleaning for NWP recovery at low NaClO concentrations

**Fig. 6:**
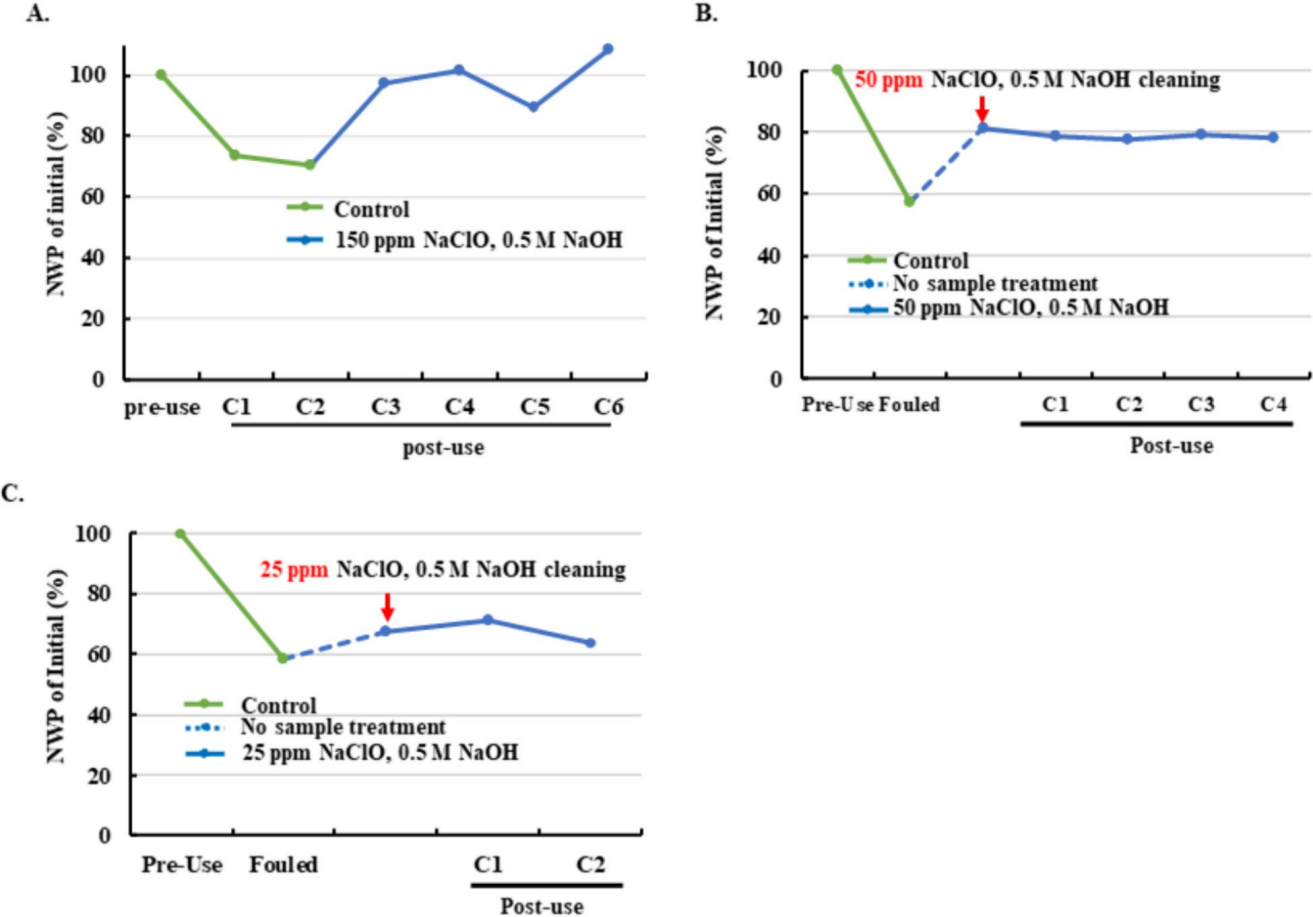
Effect of combined NaClO and NaOH cleaning on NWP recovery at different NaClO concentrations. **A**–**C** NWP profiles for membranes cleaned by traditional NaOH (green lines) and by NaClO/NaOH combinations (blue lines) at NaClO concentrations of 150 ppm (**A**), 50 ppm (**B**), and 25 ppm (**C**). Initial NWP was defined as the pre-use value before cycle 1. (NWP values up to ~ 108% were observed only after 150 ppm NaClO cleaning, attributable to transient membrane de‑compaction and enhanced wetting, together with routine temperature/flow/TMP normalization tolerance; this small upward deviation does not affect between‑group comparisons.)

Sodium hypochlorite (NaClO) is widely recognized for its cost-effectiveness and strong oxidizing capability (Cui et al. 2018). Although commonly used in water treatment, its application in biologics manufacturing is limited due to concerns about membrane integrity and chemical carryover. Previous studies have suggested that combining NaClO with NaOH enhances cleaning efficiency (Krack. 1995; Wu et al. 2006). For PES UF/DF chemical cleaning against protein fouling, published practice generally uses hypochlorite in the few‑hundred‑ppm range, with a well‑cited example at ~ 250 ppm NaClO in 0.5 M NaOH achieving multi‑cycle NWP recovery (Ahmed et al. 2006). In addition, vendor user guides allow higher levels (~ 200–500 ppm) under validated compatibility and controlled exposure (Merck 2021). To reduce potential NaClO impacts on UF/DF membranes (membrane integrity and chemical carryover), lower yet effective operating concentrations were investigated. To evaluate this approach, fouled membranes were cleaned using NaClO combined with NaOH after two UF/DF cycles. Cleaning with 150 ppm NaClO and 0.5 M NaOH restored NWP from 70% to nearly 100% of the initial value after a single application (Table 5 and Fig. 6A).

**Table 5:** Process flow for NaClO cleaning compared with the control

UF/DF steps	Control (NaOH cleaning)	NaClO + NaOH cleaning
Rinse1(HPW)	x	x
Sani. (NaOH)	x	x
Rinse2 (HPW)	x	x
Pre-use NWP testing	x	x
EQ/UF/DF/OC/recirc	x	x
Rinse3 (HPW)	x	x
Sani	x (1 M NaOH)	x (NaClO + NaOH)
Rinse4 (HPW)	x	x
Post-use NWP testing	x	x
Storage	x	x

Lower concentrations were then tested to identify a practical limit. Cleaning with 50 ppm NaClO recovered approximately 24% of initial NWP, whereas 25 ppm achieved only 9.2% recovery (Fig. 6B, C). Extended testing showed that membranes cleaned with 50 ppm NaClO maintained stable NWP over multiple cycles, while those cleaned with 25 ppm exhibited progressive decline. These results suggest that 50 ppm represents a reasonable lower limit for effective cleaning under the conditions tested. Residual NaClO after Rinse 4 and equilibration was below 0.02 ppm, indicating negligible carryover risk. Collectively, these findings demonstrate that combining NaClO with NaOH provides robust NWP recovery—even at reduced NaClO concentrations—while maintaining process consistency and minimizing chemical exposure.

### Overview of UF/DF process performance

Across all runs, the UF/DF process exhibited highly consistent operational behavior. As expected, permeate flux decreased during UF, remained stable during diafiltration, and further declined during the final over‑concentration step. Processing time and overall product yield followed similarly consistent patterns, with no meaningful differences observed between cycles or between experimental and control groups. These results confirm that the evaluated cleaning strategies did not affect UF/DF operational performance. Representative flux, processing‑time, and yield profiles are provided in Supplemental Figs. S1–S5. For all evaluated cycles, protein signals in the permeate were at trace/near‑baseline levels by NanoDrop ND‑2000 and normalized water permeability (NWP) after cleaning returned to the acceptance window without monotonic drift, indicating no loss of retention or structural deterioration under the long‑term cleaning conditions.

## Discussion

Our findings demonstrate that permeate-closed cleaning effectively mitigates fouling by enhancing hydrodynamic shear and redistributing flow within the cassette channels. In practice, this mechanism operates by promoting lateral flow and limiting cake compaction under low TMP during cleaning. This approach primarily targets loosely attached surface foulants and gel layers, reducing NWP decay without compromising process performance. Combining permeate-closed cleaning with NaOH soaking further improves cleaning efficiency, likely due to chemical dissolution of residual proteinaceous deposits that mechanical shear alone cannot remove. The combined mechanical (shear) and chemical (alkaline solubilization) actions provide complementary removal pathways. The synergistic effect of these two mechanisms explains the superior performance of the combination strategy.

High-flux permeate-closed cleaning (PCC) offers an operationally simpler alternative to forward/reverse cleaning while maintaining comparable efficiency. Increased feed flux enhances turbulence and shear, accelerating foulant detachment, while localized backpressure promotes partial backwashing. However, the acceptable negative TMP varies by membrane material and configuration; thus, PCC parameters should be optimized for different membranes and process conditions. Reverse permeate-closed cleaning provides additional benefit by cleaning regions less accessible to forward flow, but if reverse flow is impractical at scale, multiple forward PCC steps can compensate. This indicates that PCC can be tuned as a scalable lever—either via flux setpoints or step repetitions—to achieve equivalent outcomes without changing hardware flow direction.

Backwashing specifically addresses foulants embedded within membrane pores, which are less responsive to surface flushing. By reversing flow from the permeate side under controlled negative TMP, backwashing dislodges deeply trapped deposits, restoring NWP and extending membrane life. This mechanism complements PCC, which primarily acts on surface fouling. Accordingly, backwashing is most effective when triggered after surface layers have been thinned by PCC, minimizing hydraulic resistance during reverse flow.

The combination of NaClO and NaOH cleaning targets persistent organic foulants and potential biofilm residues through oxidative degradation and alkaline hydrolysis. While NaClO is highly effective, its concentration must be carefully controlled to avoid membrane damage. Our data indicate that 50 ppm NaClO combined with 0.5 M NaOH achieves substantial NWP recovery without compromising integrity, suggesting a practical lower limit under tested conditions. Given variability in polymer chemistry and support geometry, this concentration window should be verified per membrane type and screen design to balance efficacy and lifetime. However, optimal concentrations may vary depending on membrane chemistry, screen type, and process parameters.

Collectively, these results highlight that different cleaning schemes address different foulant types and mechanisms:

- PCC and F&RPCC → surface foulants and gel layers (hydrodynamic removal)
- NaOH soaking → proteinaceous residues (chemical dissolution)
- Backwashing → pore-blocking foulants (reverse flow removal)
- NaClO + NaOH → resistant organic foulants and biofilm (oxidative + alkaline action)

This mechanistic mapping provides a practical basis to assemble cleaning sequences according to the dominant foulant class present in a given run (surface layer → PCC; proteinaceous residue → NaOH; pore blockage → backwash; persistent organics/biofilm → NaClO + NaOH). Understanding these relationships is critical for designing robust, scalable cleaning protocols tailored to biologics UF/DF processes. Beyond improving cleaning efficiency, these strategies contribute to sustainable bioprocessing by reducing membrane replacement frequency and minimizing chemical usage, aligning with industry goals for resource efficiency and environmental responsibility. In operational terms, the framework supports cycle-wise decision‑making (when to add NaOH, when to insert a backwash step, when to escalate to NaClO) to maintain NWP recovery without compromising membrane integrity.

## Conclusion

This study provides a systematic evaluation of UF/DF membrane cleaning strategies for biologics manufacturing. Key findings include:

- Permeate-closed cleaning alleviates NWP decay and, when combined with NaOH soaking, offers synergistic benefits for removing both surface and residual foulants.
- High-flux PCC simplifies cleaning workflows while maintaining efficiency, making it suitable for large-scale operations.
- Backwashing effectively restores NWP in fouled membranes by removing pore-blocking deposits, extending membrane lifetime.
- NaClO + NaOH cleaning achieves robust NWP recovery even at reduced NaClO concentrations (≥ 50 ppm), minimizing chemical exposure while maintaining effectiveness.

These strategies can be flexibly integrated into UF/DF processes to address different foulant types and operational constraints. By linking cleaning mechanisms to foulant characteristics, this work provides practical guidance for optimizing membrane reuse and ensuring process consistency in industrial biologics production.

Building on these findings, future work will expand the evaluation of cleaning strategies to additional UF/DF membrane materials, enabling a broader understanding of material‑specific responses to physical and chemical cleaning. Moreover, pilot‑scale and manufacturing‑scale verification will be conducted to assess the scalability, operational robustness, and long‑term economic impact of the optimized cleaning sequences. These efforts will support the translation of laboratory‑scale insights into practical, industry‑ready cleaning solutions for biologics manufacturing.

## Supplementary Information

Additional file1 (PDF 153 KB)

Additional file1 (DOCX 14 KB)

## Data Availability

The datasets used and/or analyzed during the current study are available from the corresponding author on reasonable request.

## Abbreviations

DV: Diafiltration Volume
F&RPCC: Forward and reverse permeate-closed cleaning
HPW: Highly purified water
LMH: Liter/Square Meter/Hour
mAb: Monoclonal antibody
NaOH: Sodium hydroxide
NaClO: Sodium hypochlorite
NWP: Normalized water permeability
OC: Over concentration
PCC: Permeate-closed cleaning
PES: Polyethersulfone
TFF: Tangential-flow filtration
TMP: Transmembrane pressure
UF/DF: Ultrafiltration/diafiltration
