# Supplementary material for: Enhanced cleaning strategies for UF/DF membranes in biopharmaceutical downstream processing
Source: Bioresour Bioprocess. 2026 Apr 2;13(1):47. doi: 10.1186/s40643-026-01045-0 (PMC13046915; doi:10.1186/s40643-026-01045-0)

Supplemental Figure 1

A.

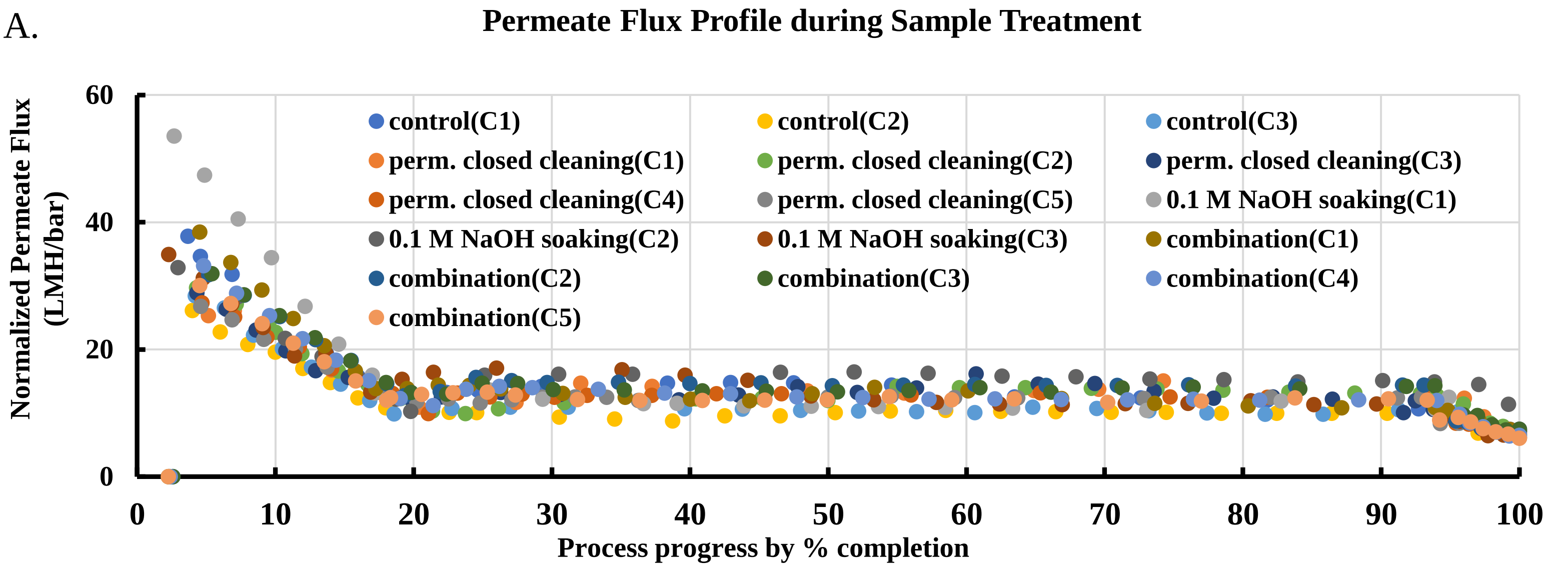

B.

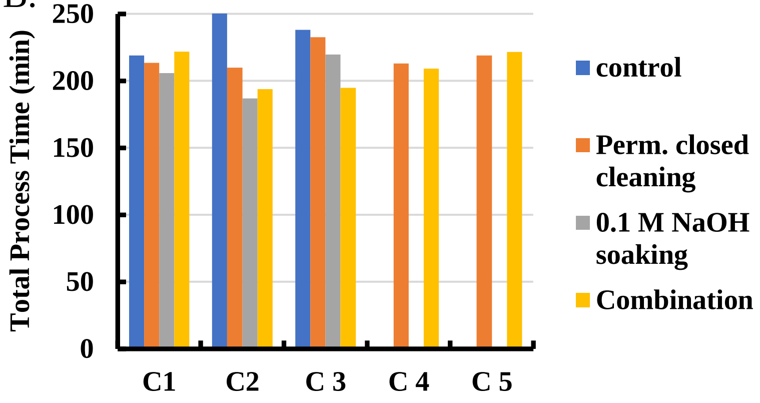

C.

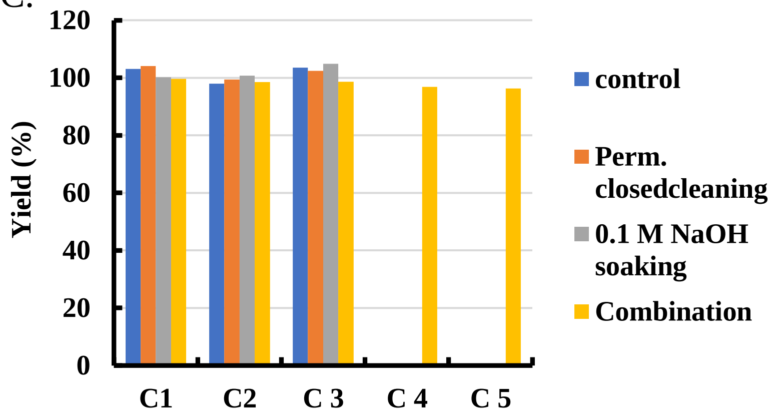

Supplemental Figure 2

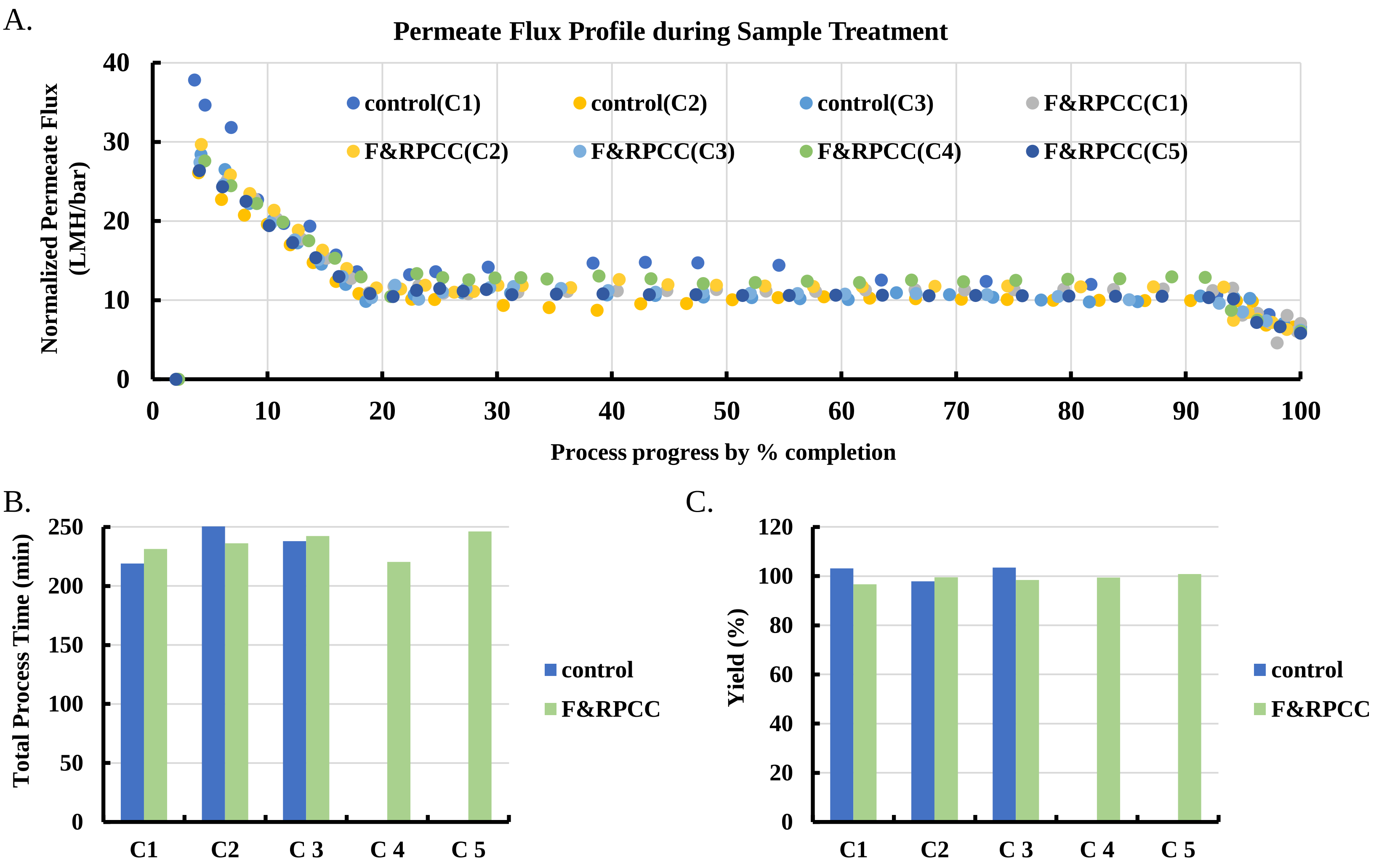

Supplemental Figure 3

A.

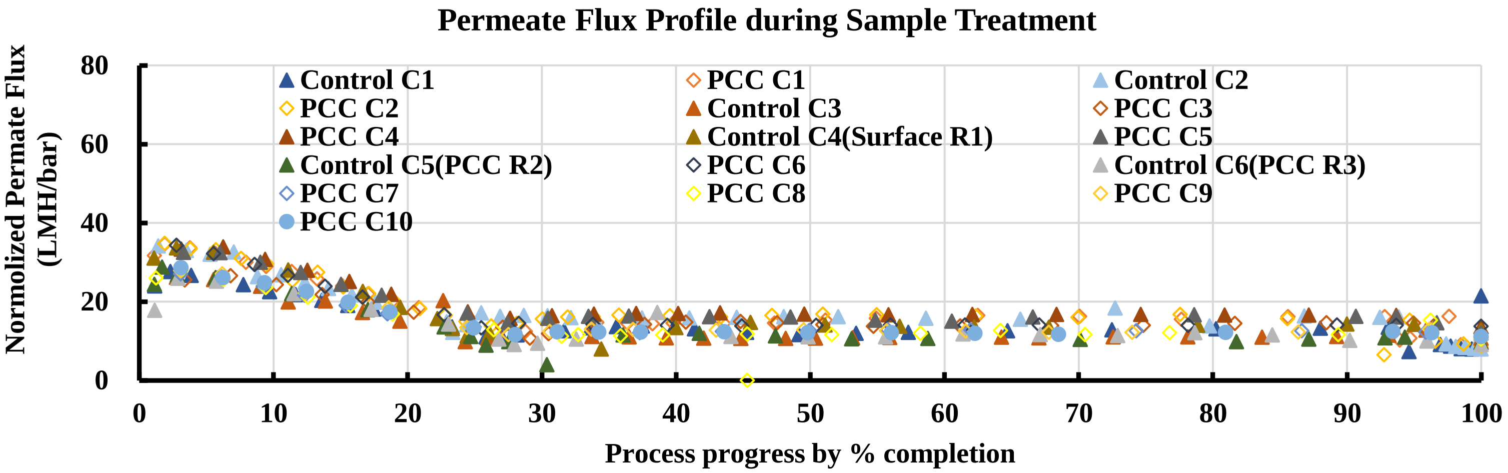

B.

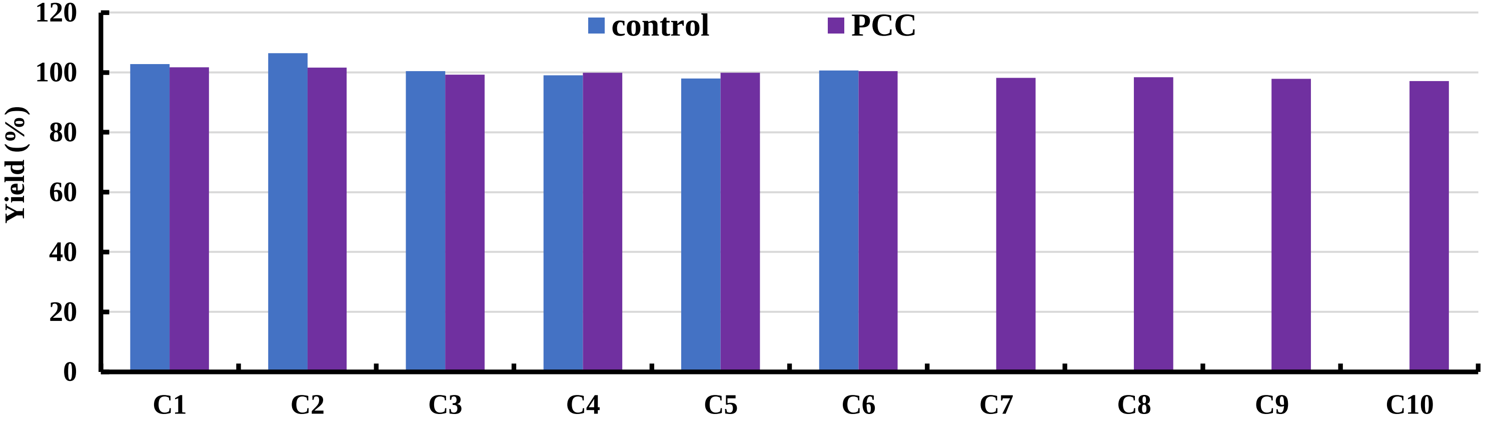

Supplemental Figure 4

A.

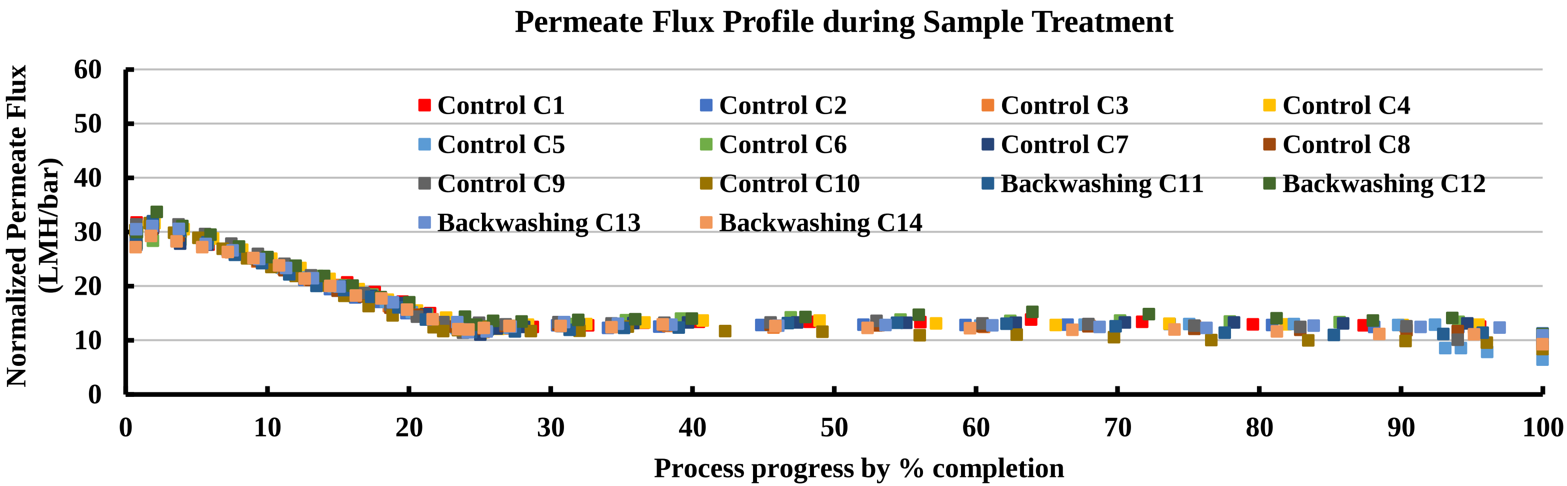

B.

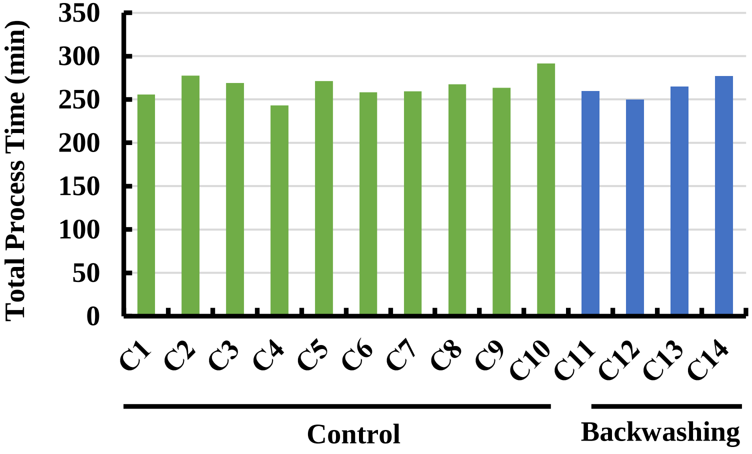

C.

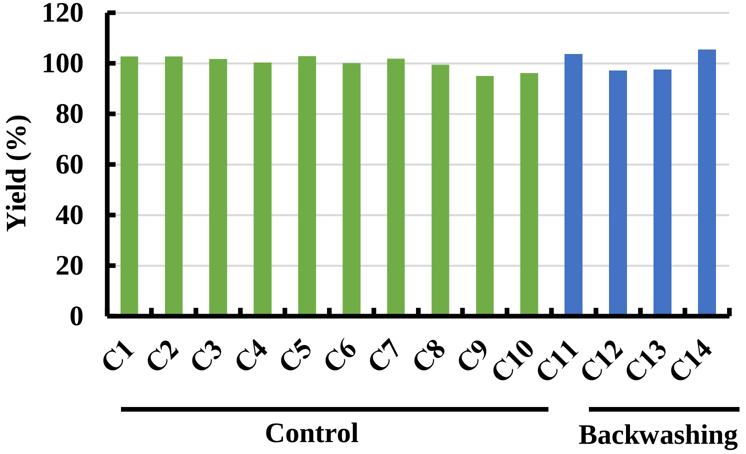

Supplemental Figure 5

A.

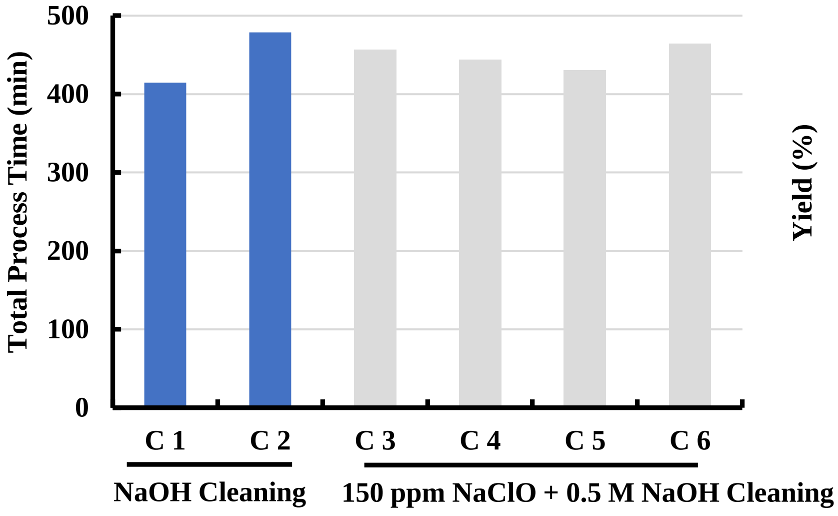

B.

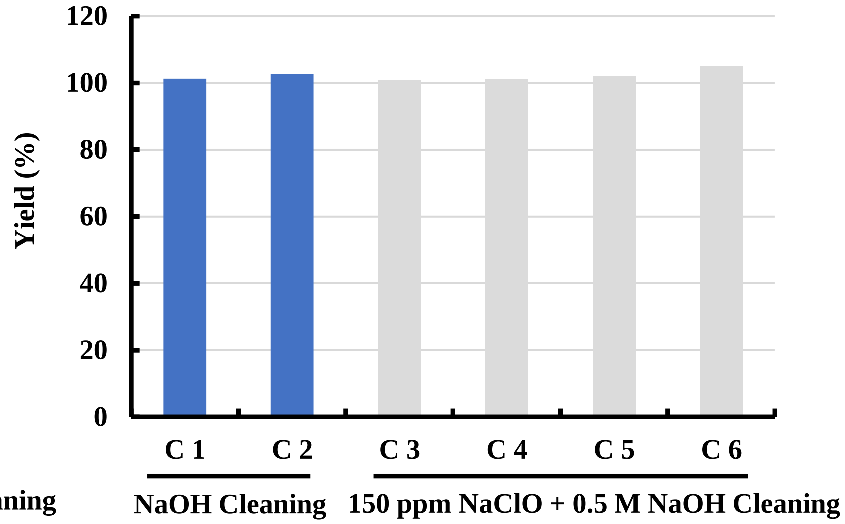

C.

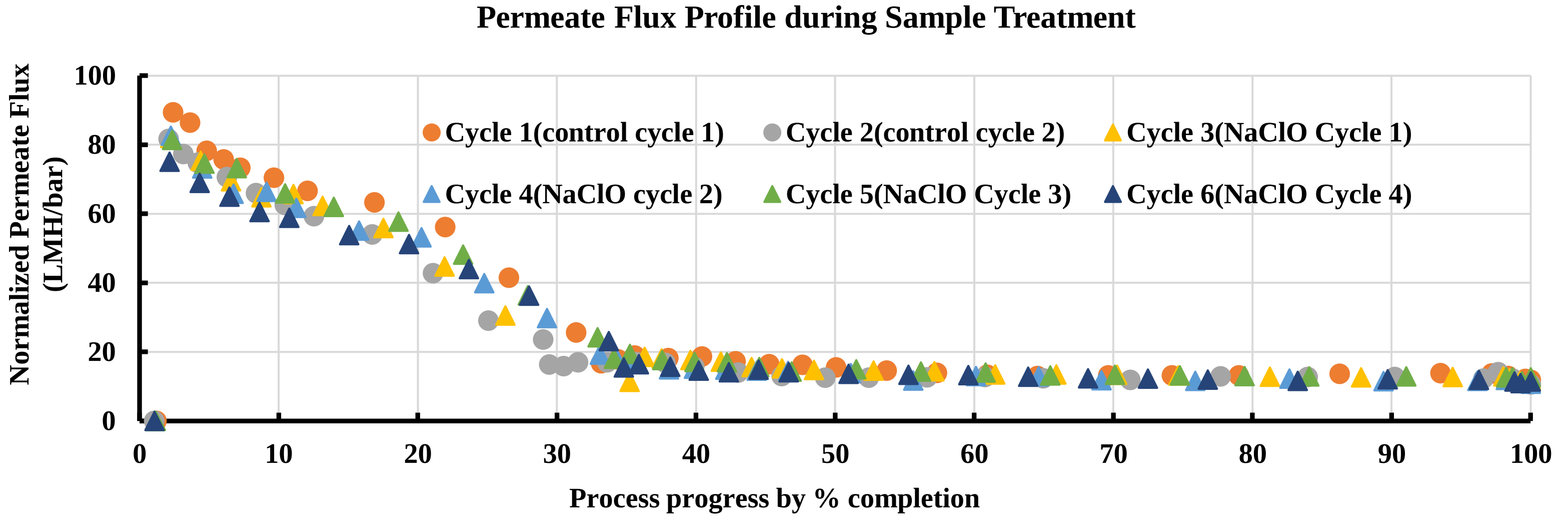

Supplement: Supplementary file 1 — Additional file1 (PDF 153 KB) [file 40643_2026_1045_MOESM1_ESM.pdf]
