# Supplementary material for: Enhanced cleaning strategies for UF/DF membranes in biopharmaceutical downstream processing
Source: Bioresour Bioprocess. 2026 Apr 2;13(1):47. doi: 10.1186/s40643-026-01045-0 (PMC13046915; doi:10.1186/s40643-026-01045-0)
Supplement: Supplementary file 2 — Additional file1 (DOCX 14 KB) [file 40643_2026_1045_MOESM2_ESM.docx]

**Supplemental Figure 1. Process performance comparison among four cleaning strategies.**
(A) Permeate flux profiles during UF/DF processing for control, permeate-closed cleaning, NaOH soaking (0.1 M), and their combination across all cycles. (B) Total process time for the four groups. (C) Product yield for the four groups.

**Supplemental Figure 2. Process performance comparison between control and F&RPCC groups.**

(A) Permeate flux profiles during UF/DF processing for control and F&RPCC across all cycles. (B) Total process time for the two groups. (C) Product yield for the two groups.

**Supplemental Figure 3. Process performance comparison between control and high-flux permeate-closed cleaning (PCC) groups.**

(A) Permeate flux profiles during UF/DF processing for control and PCC across all cycles. (B) Product yield for the two groups.

**Supplemental Figure 4. Process performance comparison between control and backwashing groups.**

(A) Permeate flux profiles during UF/DF processing for control and backwashing across all cycles. (B) Total process time for the two groups. (C) Product yield for the two groups.

**Supplemental Figure 5. Process performance comparison between NaOH cleaning (control) and NaClO/NaOH combination cleaning.**

(A) Total process time for control (1 M NaOH) and combination cleaning with 150 ppm NaClO + 0.5 M NaOH. (B) Product yield for the two cleaning methods. (C) Permeate flux profiles during UF/DF processing for control and combination cleaning across all cycles.
